# P-890. Risk Factors Associated with Carbapenem-Resistant Enterobacteria Bacteremia and Mortality in Patients with Acute Leukemia

**DOI:** 10.1093/ofid/ofae631.1081

**Published:** 2025-01-29

**Authors:** Viridiana Ruiz, Patricia Volkow-Fernández, Consuelo Velazquez-Acosta, María José Mendoza-Palacios, Beda Islas-Muñoz

**Affiliations:** Instituto Nacional de Cancerología, Ciudad de Mexico, Distrito Federal, Mexico; Instituto Nacional De CancerologÍa, Mexico City, Distrito Federal, Mexico; Instituto Nacional de Cancerología, Ciudad de Mexico, Distrito Federal, Mexico; Instituto Nacional De CancerologÍa, Mexico City, Distrito Federal, Mexico; Instituto Nacional de Cancerologia, México, Distrito Federal, Mexico

## Abstract

**Background:**

The inclusion of highly cytotoxic drugs for the treatment of acute leukemia (AL) in the last decade has exposed patients to more extended periods of severe neutropenia. The study aims to describe the prevalence and risk factors for blood-stream infection (BSI) due to carbapenem-resistant enterobacteria (CRE) in patients with AL receiving chemotherapy.

**Table 1.**
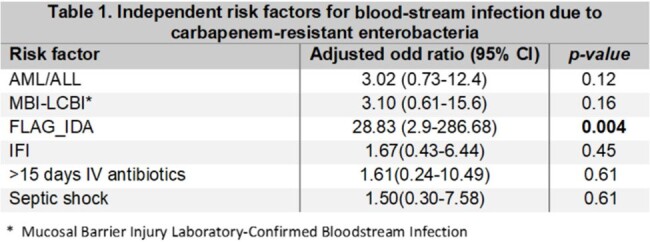
Independent risk factors for blood-stream infection due to carbapenem-resistant enterobacteria

FLAG-IDA chemotherapy regimen had an OR 28.8 (CI 2.9-286.68. p=0.004) for CRE-BSI

**Methods:**

The study was performed at a referral center for adult patients with cancer in Mexico City. From January 2021 to December 2022, all patients with AL treated from first to fourth line chemotherapy were included. CRE and non-CRE BSI were compared for categorical variables with Pearson's chi-square. A bivariate logistic regression analysis was performed to identify risk factors associated with mortality and isolation of CRE.

**Table 2.**
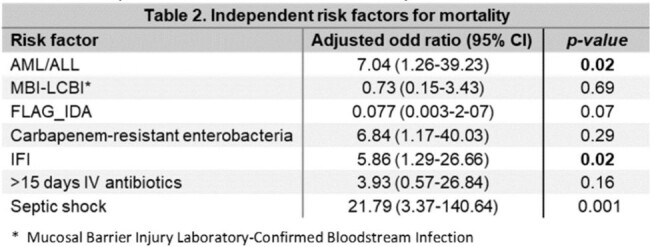
Independent risk factors for mortality Patients with AML had an OR 7 (CI 1.26-39.23 p=0.026), isolation of CRE OR 6.8 (CI 1.17-0.03 p=0.03) and being diagnosed with invasive fungal infection (IFI) OR 5.2 (CI 1.29-26.66 p=0.02) for mortality

**Results:**

One hundred ten patients were included: 62 (56.3%) with acute lymphoblastic leukemia (ALL) and 48 (43.6%) with acute myeloid leukemia (AML). 119 BSI were confirmed, 41 (34%) blood isolates were antimicrobial resistant, 23 (56.1%) had extended-spectrum beta-lactamases (ESBL), and 16 (39%) had CRE. BSIs were classified as 70 (58.8%) Mucosal Barrier Injury Laboratory-Confirmed Bloodstream Infection (MBI-LCBI), 24 (20.1%) secondary, 23 (19.3%) catheter-related and 2 (1.6%) primary. Of the 23 (56.1%) isolates with ESBL and 16 (39%) CRE, all were classified as MBI-LCBI, as were two (4.9%) methicillin-resistant *S. aureus*. Overall, 30-day mortality was 9.2%, and at 24 weeks, 16.8%.

In the bivariate analysis, receiving a FLAG-IDA had an OR 28.8 (CI 2.9-286.68. p=0.004) for CRE-BSI (Table 1). For mortality, having AML had an OR 7 (CI 1.26-39.23 p=0.026), CRE BSI isolates OR 6.8 (CI 1.17-0.03 p=0.03), and being diagnosed with invasive fungal infection (IFI) OR 5.2 (CI 1.29-26.66 p=0.02) (Table 2). Kaplan-Meier model comparing patients with and without CRE-BSI showed a mean survival of 17.8 months in CRE-BSI vs. 23.4 months without CRE-BSI, although non statistical significance (Figure 1).

**Figure 1.**
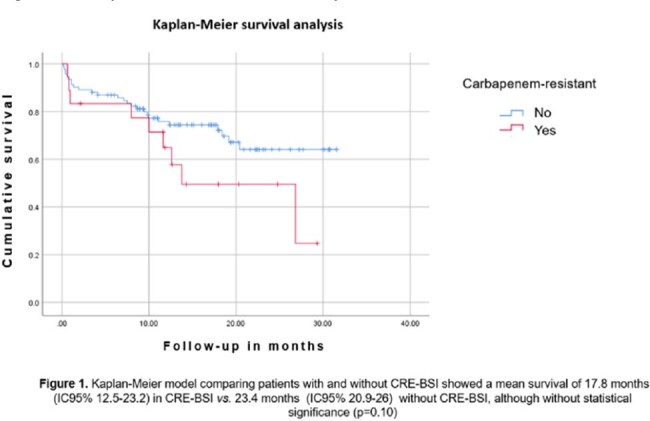
Kaplan-Meier survival analysis Kaplan-Meier model comparing patients with and without CRE-BSI showed a mean survival of 17.8 months in CRE-BSI vs. 23.4 months without CRE-BSI, although without statistical significance

**Conclusion:**

In this cohort, overall mortality was associated with AML, having CRE isolated in blood, and having been diagnosed with IFI. Having a CRE-BSI was associated with receiving FLAG-IDA. This highly myelosuppressive chemotherapy regime has become a niche for antimicrobial resistance development in our institution.

**Disclosures:**

**All Authors**: No reported disclosures

